# BMP-2 and BMP-2/7 Heterodimers Conjugated to a Fibrin/Hyaluronic Acid Hydrogel in a Large Animal Model of Mild Intervertebral Disc Degeneration

**DOI:** 10.1089/biores.2015.0025

**Published:** 2015-10-01

**Authors:** Mirte Peeters, Suzanne E.L. Detiger, Lindsay S. Karfeld-Sulzer, Theo H. Smit, Avner Yayon, Franz E. Weber, Marco N. Helder

**Affiliations:** ^1^ Department of Orthopaedic Surgery, VU University Medical Center, Amsterdam, The Netherlands.; ^2^ Center for Translational Regenerative Medicine (CTRM), MOVE Research Institute Amsterdam, Amsterdam, The Netherlands.; ^3^ University Hospital, Cranio-Maxillofacial and Oral Surgery/Bioengineering, Zürich, Switzerland.; ^4^ ProCore Biomed Ltd., Weizman Science Park, Nes Ziona, Israel.

**Keywords:** bone morphogenetic protein, fibrin/hyaluronic acid hydrogel, intervertebral disc, large animal model, regenerative medicine

## Abstract

Intervertebral disc (IVD) degeneration is etiologically associated with low back pain and is currently only treated in severe cases with spinal fusion. Regenerative medicine attempts to restore degenerated tissue by means of cells, hydrogels, and/or growth factors and can therefore be used to slow, halt, or reverse the degeneration of the IVD in a minimally invasive manner. Previously, the growth factors bone morphogenetic proteins 2 and 7 (BMP-2, -7) were shown to enhance disc regeneration, *in vitro* and *in vivo.* Since BMPs have only a short *in vivo* half-life, and to prevent heterotopic ossification, we evaluated the use of a slow release system for BMP-2 homodimers and BMP-2/7 heterodimers for IVD regeneration. BMP growth factors were conjugated to a fibrin/hyaluronic acid (FB/HA) hydrogel and intradiscally injected in a goat model of mild IVD degeneration to study safety and efficacy. Mild degeneration was induced in five lumbar discs of seven adult Dutch milk goats, by injections with the enzyme chondroitinase ABC. After 12 weeks, discs were treated with either FB/HA-hydrogel only or supplemented with 1 or 5 μg/mL of BMP-2 or BMP-2/7. BMPs were linked to the FB/HA hydrogels using a transglutaminase moiety, to be released through an incorporated plasmin cleavage site. After another 12 weeks, goats were sacrificed and discs were assessed using radiography, MRI T2* mapping, and biochemical and histological analyses. All animals maintained weight throughout the study and no heterotopic bone formation or other adverse effects were noted during follow-up. Radiographs showed significant disc height loss upon induction of mild degeneration. MRI T2* mapping showed strong and significant correlations with biochemistry and histology as shown before. Surprisingly, no differences could be demonstrated in any parameter between intervention groups. To our knowledge, this is the first large animal study evaluating BMPs conjugated to an FB/HA-hydrogel for the treatment of mild IVD degeneration. The conjugated BMP-2 and BMP-2/7 appeared safe, but no disc regeneration was observed. Possible explanations include too low dosages, short follow-up time, and/or insufficient release of the conjugated BMPs. These aspects should be addressed in future studies.

## Introduction

Low back pain has been the worldwide leading cause of years lived with disability for several decades according to the Global Burden of Disease Study 2010.^1^ Intervertebral disc (IVD) degeneration has–among other contributing factors–been established as an important etiological feature of low back pain.^2^ Standard medical care for severe painful disc degeneration currently consists of conservative therapy for pain reduction, physiotherapy, and invasive surgical procedures like spinal fusion.^3^ However, conventional clinical practice has not yet provided an elegant, minimally invasive treatment modality for disc degeneration at an early stage. Regenerative medicine aims to address disc degeneration at an early stage, where (stem) cells, extracellular matrix-supporting hydrogels, and/or growth factors are promising candidates to slow, halt, or reverse the degenerative process.^4–6^ In addition, these promising solutions can be executed using minimally invasive procedures and will therefore prevent high surgical costs while maintaining or improving quality of life.

Bone morphogenetic proteins (BMPs), a widely used group of growth factors, are anabolic, multipotent proteins that are involved in skeletal development and repair.^7,8^ Besides their role in osteogenesis, BMPs also play an important role in chondrogenesis and the maintenance of the extracellular matrix of articular and IVD cartilage.^9^ Several research groups have investigated the regenerative potential of BMPs for IVD applications, both *in vivo* and *in vitro*. BMP-2 has been demonstrated to increase extracellular matrix expression and synthesis in rat, bovine, and human IVD cells, without expression of an osteogenic phenotype.^10–14^ Similarly, BMP-7 has been shown to promote extracellular matrix metabolism in rat and rabbit IVD cells.^15,16^ Also, a beneficial effect of BMP-7 on human nucleus pulposus (NP) and annulus fibrosis (AF) cells was identified, as demonstrated by an increased cell proliferation and proteoglycan synthesis.^17^ However, although proteoglycan production increased over time, this effect was slower than observed in rabbit and bovine NP and AF cells receiving a similar dose of BMP-7.^15,18^ *In vivo,* intradiscal injection of adenoviral vectors carrying the BMP-2 gene slowed down IVD degeneration in a rabbit annular puncture model.^7^ In addition, injection of BMP-7 increased disc height and proteoglycan content in both an annular puncture and enzymatically degenerated disc rabbit model.^5,19^

BMP-2 has also been applied for lumbar interbody fusion to induce bone formation, but this has led to severe adverse effects, including heterotopic ossification, retrograde ejaculation, and possible increased risk of malignancy.^20–22^ Heterotopic bone formation following lumbar spinal fusion supplemented with BMP-2 or BMP-7 is described in several studies with varying incidence rates.^23–26^ A systematic review in 2010 reported a mean rate of 8% (range 0–75%) for heterotopic bone formation associated with BMP use in lumbar spine surgery.^22^ In this study, it should be mentioned that the studies in which these complications were reported used high doses of BMPs (3.5–12 mg BMP per treated level).

To enhance IVD regeneration but avoid heterotopic ossification outside of the IVD, slow release drug delivery systems may be applied. Moreover, the delayed regenerative inductive response of human IVD cells to BMP-7 and the short *in vivo* circulation times of BMPs imply an advantageous effect of a prolonged exposure to BMPs.^27^ One suitable slow release system utilizes covalent incorporation of BMPs using transglutaminase (TG) crosslinking into a hydrogel of fibrin and hyaluronic acid (FB/HA). This FB/HA hydrogel has previously been shown to support NP-cell function and restores disc height and compressive stiffness *ex vivo*.^28^ *In vitro* experiments demonstrated that covalently bound BMPs can be retained in fibrin hydrogels until released by cells through an included plasmin-cleavable site.^29,30^ Treatment of a critical-size cranial defect in rats with this BMP-2 coupled to a fibrin gel induced 76% more bone formation compared to the wild-type recombinant human BMP-2 (rhBMP-2).^29^ A recent study reproduced these findings and also demonstrated that the BMP-2/7 heterodimer conjugated to a fibrin hydrogel was more efficacious compared with the commonly used BMP-2 homodimer in a similar rat calvarial defect.^30^

This study evaluated the safety and efficacy of a novel slow release BMP technology for IVD regeneration in a previously validated goat IVD degeneration model.^31^ After induction of mild IVD degeneration with chondroitinase ABC (CABC), intradiscal injections of 1 or 5 μg/mL of either BMP-2 or BMP-2/7, covalently conjugated to the FB/HA hydrogel, were performed. After a follow-up time of 12 weeks, safety and efficacy were assessed using multiple outcome parameters (radiography, MRI T2* mapping, and biochemical and histological analyses). Besides a dose–response effect, this setup also allowed a comparison in effectiveness between the BMP-2 homodimer and the BMP-2/7 heterodimer for IVD regeneration.

## Materials and Methods

### Animals and surgical procedure

Both the Scientific Board and the Animal Ethics Committee of the VU University Medical Centre approved the research protocol. Seven skeletally mature female Dutch milk goats (average age: 3.8 ± 1.5 years) with an average weight of 74 ± 11 kg were used for this study. All goats underwent two surgeries: in the first procedure, lumbar IVDs (L1L2–L5L6) of each goat were injected with 0.25 U/mL CABC dissolved in PBS using a 29G needle, whereas another disc (T13L1) was left as a healthy control. CABC cleaves proteoglycans and thereby mimics disc degeneration, as validated previously in our group.^31^ During the second procedure, 12 weeks later, IVDs were injected with the FB/HA hydrogels supplemented with either BMP-2 or BMP-2/7 in two concentrations or with hydrogel only (vehicle control), prepared as described below. After the second surgery, goats were monitored for 12 weeks at which point they were sacrificed, and lumbar spines (T13–L6) where harvested for further analysis.

### Production of BMPs and crosslinking to a hydrogel carrier

**FIG. 1.**
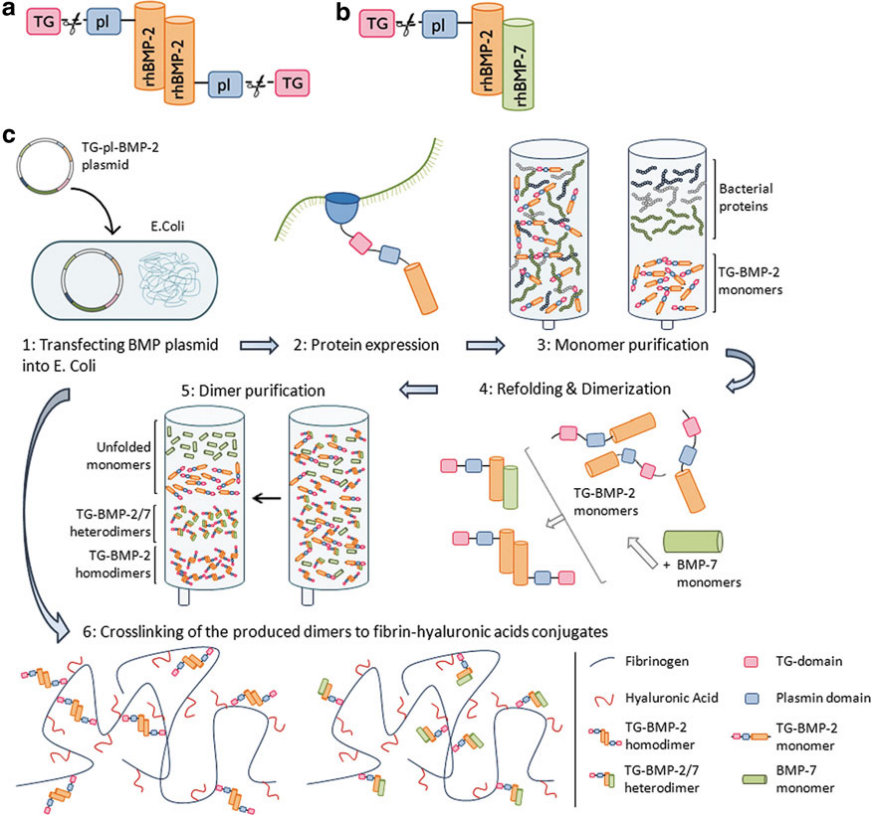
**(a)** Schematic overview of the engineered TG-BMP-2 homodimer, containing two transglutaminase (TG) crosslinking sites and two plasmin (pl) cleavage sites. **(b)** Schematic overview of the engineered TG-BMP-2/BMP-7 heterodimer. **(c)** Production of the TG-BMP2 homodimer and TG-BMP2/7 heterodimer. Starting with the engineering of a BMP-2 plasmid vector containing two extra-amino acid sequences; TG for crosslinking the BMP-2 onto the fibrin and a plasmin site to allow enzymatically cleaving off the growth factor from the fibrin.

In the present study, a fibrinogen/hyaluronic acid (FB/HA) conjugate was used as a carrier for the BMP-2 and the BMP-2/7 dimers. rhBMP-2 containing extra-amino acids for TG crosslinking and plasmin cleavage sites (pl) at the N terminus was produced as described previously.^29,32^ Briefly, the DNA encoding for the *TG* and *pl* amino acids were cloned into a plasmid containing the genetic sequence for rhBMP-2 (pET23aBMP2 vector) and transfected into the E. coli bacteria. TG-pl-BMP-2 monomers were produced with standard protein expression procedures. Obtained monomers were purified through affinity and size exclusion chromatography, subsequently refolded, and in a final purification step, dimers were separated from monomers and unfolded growth factors (Fig. 1). For the production of the TG-pl-BMP-2/BMP-7 heterodimer, rhBMP-7 was added during refolding and dimerization (Fig 1, step 4). Final concentrations of the produced TG-pl-BMP-2 proteins and TG-pl-BMP-2/BMP-7 were determined. The engineered fused growth factors are henceforth mentioned as TG-BMP-2 and TG-BMP-2/7. FB/HA conjugates were synthesized by the reaction of a buffered fibrinogen solution with a HA-active ester solution using HA with a molecular weight of 235 kDa, and the ratio FB/HA was 17:1(ProCore Bio Med Ltd.).^28^ TG-BMPs were mixed in a thrombin solution (5.2 U/mL). Just before injection, FB/HA conjugates and the TG-BMP-containing thrombin solution were mixed and injected into the IVD before polymerization. For each TG-BMP, two different concentrations were used, with a final concentration of TG-BMP in the hydrogels of 1 or 5 μg/mL.

### Radiological analysis

Before both surgeries and autopsy, standardized lateral lumbar radiographs were obtained. For each IVD, a disc height index (DHI) was calculated as previously described.^33^ In short, the height of the IVD and adjacent caudal vertebral body were measured by averaging three measurements. DHI was then calculated by dividing the average IVD height by the vertebral body height, thus correcting for interanimal size differences.

### Magnetic resonance imaging

MRI scans were acquired from all lumbar spines within 2–3 h after autopsy using a 1.5T MR scanner (Magnetom Symphony, Syngo MR VA30; Siemens Healthcare). Sagittal scans were performed using a T2-weighted turbo spin echo (TSE) sequence, followed by a multiecho gradient echo sequence for T2* mapping (echo times of 5.7, 10.9, 16.05, 21.2, and 26.4 msec) as recently described and validated in our group.^34^ T2* relaxation times were calculated by fitting the signal intensities of the five echo times, for five regions of interest (ROIs) covering the IVD from anterior to posterior. The ROIs were drawn in a similar manner for all discs, according to a proportional division; ROIs 1 and 3 covered 27.5% of total disc diameter, whereas ROIs 2, 4, and 5 each covered 15%. This way ROIs 1, 3, and 5 represent the anterior AF, NP, and posterior AF, respectively. ROIs 2 and 4 represent the transition zones between NP and AF and were not used for further analysis. By copying the ROIs drawn in the first series into the subsequent series, the ROI size and position were kept equal for all echo times. T2* relaxation times were calculated by fitting the echo time intensities using a linear-log least-squares method using Microsoft Excel (Microsoft^®^ Office 2010).

### Histological evaluation

After obtaining MR images, all lumbar IVDs (T13L1–L5L6), including endplates were dissected from the spine and a 4 mm paramidsagittal slice was obtained using a band saw (Exakt, Norderstedt, Germany). Sections were fixed in 4% formaldehyde, decalcified in Kristensen's fluid, cut into 3 μm thin sections using a microtome, and stained with hematoxylin and eosin and Alcian Blue-Periodic Acid Schiff. Sections were analyzed using light microscopy, and degeneration grading was scored by two blinded independent researchers on different parameters optimized for goat IVD degeneration, as described and validated previously.^33^ Differences in scoring between the observers were resolved by consensus, resulting in a final histological score ranging between 0 (healthy) and 6 (severely degenerated disc).

### Biochemical analysis

Tissue samples for biochemical analysis were obtained from an adjacent slice to the histological section; anterior AF, NP, and posterior AF were harvested, that is, from MRI ROIs 1, 3, and 5, respectively. Samples were freeze-dried (*speedvac*) and subsequently digested in 1.5 mL papain-digestion buffer containing 0.1 M sodium acetate, 0.01 M L-cysteine, and 0.01 M EDTA, after which pH was titrated to 6.6 using 1 M NaOH and finally 0.33% (w/v) papain was added to the solution (all Merck Millipore). Samples were digested overnight in a continuously shaking water bath at 65 °C. Papain digestion solutions were diluted and glycosaminoglycan (GAG) content was analyzed using a DMMB assay (Biocolor Ltd.) according to the manufacturer's protocol. The collagen content, expressed as the total amount of hydroxyproline (HYP), was quantified using a DMBA hydroxyproline assay, as described by Paul et al.^35^ Measured amounts of GAG and HYP content were expressed in micrograms per milligram tissue dry weight.

### Statistical analysis

The results of the DHI were analyzed using a Wilcoxon signed-rank test. Outcome parameters were compared and analyzed by a one-way analysis of variance (ANOVA) using a Tukey *post hoc* test for parametric data and the Kruskal–Wallis test for nonparametric data. Linear regression analyses between MRI T2* relaxation times and biochemical content and histological grades were performed. Correlations between variables were analyzed for statistical significance using Spearman's rho (ρ) coefficients. Correlations were considered strong for ρ > 0.7, moderate for 0.5 < ρ ≤ 0.7, weak for ρ ≤ 0.5, and significant for *p* < 0.05. All data were also analyzed using linear mixed models, where goat was included as a random factor. Bonferroni's *post hoc* testing was used to compare the means of different outcome parameters between experimental groups. Differences were considered statistically significant for *p* < 0.05. Statistics was performed using SPSS version 20.0 (SPSS Institute) or GraphPad Prism 6 (GraphPad Software, Inc.).

## Results

After both surgeries, all goats recovered well and showed normal ambulatory activities on the first postoperative day and body weight was maintained during follow-up. We found no significant differences in injected volumes of CABC (*p* = 0.33) between all experimental groups. The treated IVDs received an equal volume of injected vehicle or TG-BMP-hydrogel combinations (*p* = 0.59) (Table 1). This indicates a comparable CABC induction of mild degeneration and subsequent TG-BMP treatment for all intervention groups.

**Table 1.** **Injected Volumes per Experimental Group**

	Volume	
Intervention groups	CABC (μL)	BMP/vehicle (μL)
Healthy vehicle	130 ± 54	117 ± 37
TG-BMP-2 1 μg/mL	126 ± 29	151 ± 66
TG-BMP-2 5 μg/mL	116 ± 32	131 ± 32
TG-BMP-2/7 1 μg/mL	124 ± 37	120 ± 50
TG-BMP-2/7 5 μg/mL	150 ± 29	143 ± 51

Volumes of CABC and BMP or vehicle are represented as mean ± SD.

BMP, bone morphogenetic protein; CABC, chondroitinase ABC; TG, transglutaminase.

### Radiological analysis

Injection of CABC into the IVDs resulted in a significant decrease (*p* = 0.002) of 6% in DHI compared to initial DHI, representing one of the initial symptoms of IVD degeneration. Radiographs, taken before the second surgery, revealed a fractured endplate in one IVD; this disc was excluded from further analysis on the effect of the BMPs. Calcification of the NP was observed in one other IVD, prior the first surgery, as shown on radiographs and MRI scans, this disc was not used in the T2* relaxation time analysis for the different intervention groups. Radiographs and MRI scans did not reveal any osteophyte formation or other endplate irregularities.

### Effect of TG-BMPs

To assess regeneration following the injection of BMPs into the NP, many different parameters were analyzed. First, MRI T2* maps showed a significant difference in the relaxation times calculated for the NP (51.7 ±14.5 msec) compared to anterior (20.0 ± 1.9 msec) and posterior annulus fibrosus (18.1 ± 4.3 msec) (*p* < 0.0001). We did not find a regenerative response, based on the MRI T2* relaxation times, initiated by the administration of the TG-BMP-2 and TG-BMP-2/7 conjugated to a hydrogel carrier (*p* = 0.67). As early regeneration will start in the NP, the results showed focus mainly on the NP (Table 2). In line with the results found for the T2* relaxation times, we did not find any significant differences in the biochemical composition of the NP, as measured by the amount of glycosaminoglycans (GAG) and collagen content (HYP) between all different treatments (GAG, *p* = 0.15 and HYP, *p* = 0.32).

**Table 2.** **Overview of Analyzed Parameters for Each Experimental Intervention for the Nucleus Pulposus and Statistical Analysis**

	T2^*^ relaxation time (msec)	GAG content (μg/mg DW)	HYP content (μg/mg DW)	Histological grading
Control				
Mean	58.31	366.51	26.44	1.75
SD	16.51	84.86	9.82	1.28
*n*	8	8	8	8
Vehicle				
Mean	54.81	290.09	29.2	2.57
SD	17.27	69.48	8.67	1.4
*n*	6	7	7	7
TG-BMP-2 1 μg/mL				
Mean	46.99	274.74	34.71	2.17
SD	13.96	77.18	8.09	0.75
*n*	6	6	6	6
TG-BMP-2 5 μg/mL				
Mean	48.4	283.56	30.67	2.29
SD	13.35	52.28	10.71	1.5
*n*	7	7	7	7
TG-BMP-2/7 1 μg/mL				
Mean	51.91	291.21	34.16	1.86
SD	16.31	62.53	10.97	1.57
*n*	7	7	7	7
TG-BMP-2/7 5 μg/mL				
Mean	48.34	293.16	25.71	2.57
SD	10.51	66.54	3.24	1.27
*n*	7	7	7	7
Average				
Mean	51.66	302.06	29.95	2.19
SD	14.49	72.93	9.13	1.29
*n*	41	42	42	42
ANOVA	*p* = 0.67	*p* = 0.15	*p* = 0.32	*p* = 0.74

DW, dry weight; GAG, glycosaminoglycan; HYP, hydroxyproline; TG-BMP-2/TG-BMP-2/7, the covalently bound growth factors BMP2 and BMP-2/7 heterodimer to the fibrin/hyaluronic acid hydrogel through a transglutaminase moiety (TG).

Histological grading of the IVDs revealed severe degeneration in three IVDs. One of these discs concerns the IVD with a fractured endplate, which was observed before the second surgery and can therefore not be attributed to the injection of the TG-BMPs. Another severely degenerated disc is part of the negative control group and thus did not receive any TG-BMPs. Apart from one IVD, no severe degeneration in the TG-BMP groups was observed, suggesting that the doses TG-BMPs used in this study can be safely administered to the IVD. Mean histological score for all discs was 2.19 (±1.3) and no significant differences were observed comparing all interventions (*p* = 0.74).

We found large interanimal differences in the different analyzed parameters, and further evaluation using ANOVA revealed a significant effect of the factor goat for the HYP content, MRI T2* relaxation times, and histological grading. From earlier studies we know these variations can nullify the effect of the actual outcome parameters. Therefore, we performed additional analysis using linear mixed models with goat as a random factor, which did not reveal any significant differences between the applied interventions either (HYP content *p* = 0.07, T2* relaxation times *p* = 0.37, histological grading *p* = 0.51).

### Correlations

**FIG. 2.**
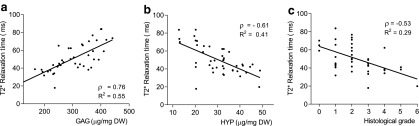
Scatter plots and linear regression lines indicating correlations between MRI T2* relaxation times (msec) and **(a)** glycosaminoglycan (GAG) content (μg/mg dry weight) (*ρ* = 0.76, *p* < 0.0001), **(b)** collagen (HYP) content (μg/mg dry weight) (*ρ* = −0.61, *p* < 0.0001), and **(c)** histological grade (range 0–6) (*ρ* = −0.53, *p* = 0.0002). ρ, Spearman's rho correlation coefficient; R^2^, linear regression coefficient.

Figure 2 shows the scatter plots and correlations between the MRI T2* relaxation times in the NP and the amount of GAG, collagen content (HYP), and histological grading. We found a strong correlation between the T2* relaxation times and the amount of GAGs for the NP (ρ = 0.76, *p* < 0.0001). Moderate, yet highly significant, correlations were observed between T2* relaxation times of the NP and the collagen content (ρ = −0.61, *p* < 0.0001) and histological grading (ρ = −0.53, *p* = 0.0002). Concerning the inner and outer annulus fibrosus, only weak and no significant correlations were found for T2* relaxation times and other outcome parameters.

## Discussion

In this study, we sought to establish the safety and efficacy of a newly synthesized slow-release delivery system for TG-BMP-2 and TG-BMP-2/7 for IVD repair in a goat model of mild IVD degeneration. This slow-release system, TG-BMP conjugated to a FB/HA hydrogel carrier, appeared to be safe, as demonstrated by the absence of any adverse effects like osteophytes, heterotopic bone formation, or inflammation. Where earlier *in vivo* studies showed the feasibility of restoring degenerated IVDs by injecting BMP-2 or BMP-7 intradiscally^36,37^ or delaying the degeneration process using adenoviral vectors carrying the BMP-2 gene,^7^ this regenerative effect was not observed in our goat IVDs. We observed no statistically significant differences between any of the treatment groups, while the individual outcome parameters correlated well with each other. The good correlations found indicate an accurate analysis of the different parameters and are in line with the correlations found previously within our group.^34^

The absence of regenerative effects may have several causes. First, the TG-BMP-2 and TG-BMP-2/7 dosages may have simply been too low for a large animal. We injected absolute amounts of maximal 1 μg TG-BMP-2 or TG-BMP-2/7 in our goat model, based on previous studies by our group, that is, a bone regeneration study in rat cranial defects.^29,30^ Other *in vivo* studies, investigating IVD regeneration, used 2-100-fold higher dosages of BMP. Injection of 7.5 μg rhBMP-2 in a rat tail disc degeneration model showed an improved MRI disc grade, using a modified Pfirrmann grade, when injected 4 weeks postannular puncture.^36^ BMP-7 was evaluated in a rabbit degeneration model using either 2 μg rhBMP-7 in 10 μL saline^37^ or 100 μg rhBMP-7 in 10 μL lactose.^19^ In a very recent study, Willems et al.^38^ injected an absolute dose of 2.5, 25 or 250 μg rhBMP-7 per IVD in a canine model of spontaneous IVD degeneration, also based on positive outcomes in prior *in vitro* studies. However, like in our study, no regenerative signs were observed. Their possible explanation of the discrepancy between the *in vitro* and the *in vivo* findings is the difference in addition frequency: biweekly for *in vitro* use versus a single dose *in vivo.*

All these studies used a wild-type rhBMP, which is not conjugated to a carrier and may therefore show a faster release and concomitantly a shorter efficacy period, compared to the conjugated TG-BMP we used for our study. This is substantiated by previous *in vitro* experiments showing a 60% retention of the TG-BMP-2 conjugated to fibrin after 50 twelve-hour wash steps with Tris-buffered saline, compared to 27% retention for the wild-type rhBMP in fibrin.^29^ Moreover, a fourfold increase in cumulative release of rhBMP-2 was found relative to both TG-BMP-2 and TG-BMP-2/7 dimers after a culture period of 2 weeks.^30^ We therefore postulate that the longer sequestration of the BMPs by the covalent conjugation to the hydrogel well compensates for the lower amounts we used in comparison to most other *in vivo* studies. Finally, the amounts used in this study and the sequestration to the fibrin hydrogel were chosen to avoid reported risks of (extradiscal) osteogenic induction, when applying high dosages of BMPs. In a study by Huang et al., injection of 100 μg rhBMP-2 in a rabbit annular tear degeneration model showed inflammatory infiltrates, increased vascularity, osteophyte formation, and endplate hypertrophy.^39^ These findings point more toward spinal fusion, which we aimed to avoid. Moreover, diffusion of nonsequestered rhBMP-7 out of the IVD may cause adverse effects. This was demonstrated in the before mentioned study of Willems et al.,^38^ in which injection of the two highest dosages rhBMP-7 resulted in extensive extradiscal bone formation. Thus, this further advocates the use of a sequestration/slow release system.

Besides the explanations mentioned earlier, our follow-up time of 12 weeks could be too short for any quantifiable, regenerative changes to occur. In the studies mentioned previously, follow-up time varied from 6 till 24 weeks post-BMP administration; however, it is well known that repair mechanisms may be faster in small animal models.^40^ The 12-week follow-up for our study was chosen to be able to detect a dose–response effect of the differently administered concentrations, TG-BMP, before a plateau phase was reached where results would be similar for all TG-BMP treatment intervention groups.

Another hypothesis may be that the absence of a regenerative effect is due to ineffective cleavage of the TG-BMP from the hydrogel carrier because of a lack of plasmin or molecules with the same function in the IVD. The engineered TG-BMP fusion protein comprises a proteolytic, plasmin-sensitive cleavage domain to allow slow release from the FB/HA hydrogel by local enzymatic activity.^29^ Although plasmin is only found in IVDs with a fractured endplate,^41^ suggesting blood-borne instead of locally synthesized plasmin, there is an upregulation of other extracellular matrix degrading proteins like MMPs and ADAMTS in the mildly degenerated IVD.^35,42^ Fibrin is one of the substrates for MMPs and we hypothesized that the upregulation of the MMPs would degrade the FB/HA hydrogel and thereby release the TG-BMPs in the NP.^43,44^ Moreover, the TG-BMP-2/7 heterodimer, conjugated to an FB/HA hydrogel, showed an increase in glycosaminoglycan synthesis of *in vitro* cultured bovine NP cells with an increasing concentration of TG-BMP-2/7. No plasmin was added to the culture medium, indicating activity of the TG-BMP without active cleaving of the plasmin domain (unpublished results FP7 Project “NPmimetic”). Prompted by the absence of regenerative effects, we aimed to assess whether the TG-BMPs were still retained in the NP at the time of sacrifice. However, due to too low sensitivity of assay methods for *in vivo* detection of the TG-BMPs, we could not properly address this question.

To our knowledge, this is the first study beyond small animal models evaluating conjugated BMPs for mild DD treatment; no large animal or clinical studies have been reported so far. One major drawback of the preceding *in vivo* studies, all performed in rodent and rabbit models, is that these animals retain their notochordal cells in adult life, whereas in humans and in goats, the notochordal cells have disappeared at the time of adolescence.^45,46^ The notochordal cells, also regarded as NP precursor cells, are thought to play a role in the maintenance of the extracellular matrix in the NP and coordinate the function of the chondrocyte-like NP cells.^47^ The presence of these cells could therefore overestimate the effect of the regenerative therapies tested. As notochordal cells are not present in the IVDs of a skeletally mature goat, we classify the goat as a more suitable animal model to study regenerative therapies.

Future studies should compare the conjugated TG-BMPs with the nonconjugated wild-type BMP-2 and BMP-2/7 for regeneration of the IVD in a large animal model, with longer follow-up times and including higher dosages of TG-BMPs. Additional gene expression analysis by real time-PCR could be performed to observe a possible shift from a catabolic to a more anabolic gene expression pattern.

## Conclusion

To our knowledge, this is the first large animal study evaluating BMPs, conjugated to an FB-HA hydrogel, for the regeneration of mildly degenerated IVDs. We showed that a slow release BMP-2 and BMP-2/7 system for IVD regeneration in goats, using engineered TG-BMP-2 and TG-BMP-2/7 proteins, can be applied safely in our goat model. However, no effect on disc regeneration was observed, as demonstrated by the absence of statistically significant differences between any of the intervention groups. Possible explanations are too low dosages of the TG-BMPs, the absence of notochordal cells, short follow-up time, and/or insufficient release of the conjugated TG-BMPs from the injected hydrogel. Future studies should compare the TG-conjugated BMPs with the nonconjugated rhBMP-2 and BMP-2/7 for regeneration of the IVD in a large animal model, with additional (longer) follow-up times and including higher dosages of TG-BMPs.

## Abbreviations Used

AF: annulus fibrosis
ANOVA: analysis of variance
BMP: bone morphogenetic protein
CABC: chondroitinase ABC
DHI: disc height index
DW: dry weight
FB: fibrin
GAG: glycosaminoglycan
HA: hyaluronic acid
HYP: hydroxyproline
IVD: intervertebral disc
MRI T2*: MRI technique, a new method for the clinical assessment of IVD degeneration
NP: nucleus pulposus
pl: plasmin
rhBMP: recombinant human BMP
ROI: region of interest
TG: transglutaminase
TSE: turbo spin echo
